# Development and Evaluation of the Course on Global Health Nursing for Indian Nursing Students

**DOI:** 10.3390/ijerph19041978

**Published:** 2022-02-10

**Authors:** Vasuki Rajaguru, Jina Oh, Mihae Im

**Affiliations:** 1 Department of Healthcare Management, Graduate School of Public Health, Yonsei University, Seoul 03722, Korea; vasuki@yuhs.ac; 2 College of Nursing, Institute of Health Science, Inje University, Busan 47392, Korea; 3 Department of Nursing, Choonhae College of Health Sciences, Ulsan 44965, Korea; mihae1219@gmail.com

**Keywords:** global health, Global Health Nursing, knowledge, skills, education intervention

## Abstract

The purpose of this study was to develop and evaluate the effectiveness of the course on Global Health Nursing for Indian nursing students, intended to introduce the course as a selective course in the nursing curriculum. Methods: A quasi-experimental nonequivalent control group pre- and post-test design was carried out. The study participants were fifty final grade nursing students, considered as an experimental (n = 25) and control group (n = 25). The participants recruited by purposive sampling were fifty 4th grade nursing students (experimental 25, control 25) at St. X College of Nursing, India. The course on Global Health Nursing was developed by using the ADDIE model, and the duration of the course was about 16 h over three weeks. The course was implemented based on Gagné’s instructional method. Data were collected by self-perceived scales of Global Health Nursing before and after the course, the participants’ opinions, and feedback providing comments about the course. Results: The study results showed improvement in knowledge (F = 8.48; *p* < 0.001), skills (F = 96.14; *p* < 0.001), and performance on Global Health Nursing (F = 5.50; *p* < 0.001) and was statistically significant. Moreover, the participants described that they were satisfied with the quality of teaching–learning and achieved the learning goals. Conclusion: The outcome of this study could be extended to the existing nursing curriculum and would be modified to formulate a standard module in the curriculum of the Indian nursing system. The study offers implications for different fields of nursing, including nursing education, research, and practice in India.

## 1. Introduction

“Global health” has become a major concern of world leaders, and issues have led to health transcending national borders and becoming more diverse in the world [1]. Koplan and colleagues suggested that global health should be an area of study, research, and practice for improving health and achieving health for all people worldwide [2]. Global health emphasizes transnational health issues, determinants, and solutions as an area for academic research and practice that prioritizes achieving health equity for all people around the world [2].

Numerous studies have recognized the important contributions of nurses and midwives to global health, including a resolution related to the strengthening of nursing and midwifery “as a means of achieving better health for all communities”; especially notable is the record of World Health Organization (WHO) and World Health Assembly (WHA) [3,4,5,6]. Moreover, Carlton emphasized global health that could be integrated into the nursing curriculum and highlighted global efforts to enhance nursing education worldwide [7]. A national study in the United States reported that all nursing professionals needed to come up with new ideas to integrate the evolving global health concepts and issues in the nursing curriculum and to integrate global health concepts into the nursing curriculum [7,8]. The Honor Society of Nursing, Sigma Theta Tau International (STTI), announced the creation of the Global Advisory Panel on the Future of Nursing to establish a voice and vision for the future of nursing and midwifery that will advance global health [9].

A study reported that both developed and developing countries must evaluate their own education assets and focus on the global health that would be most effectively implemented and measured [10]. However, there have been few attempts to grasp an overview of the consequences of global health in low-income and middle income such as developing countries as scholarly knowledge [11,12,13,14,15,16,17,18]. India is also one of the developing and middle-income countries where nursing professionals need to acquire global health knowledge in order to prepare for global competence [17]. Global Health Nursing education is recognized as a broad subject, with a variety of current teaching and learning strategies and different training standards [11,12,13]. Educators were asked to carefully consider the content and delivery methods for the introduction of global health-based subjects into the nursing curricula [16], as it is essential to offer culturally appropriate education to nursing students [7,8,9,13,14]. 

In the Indian context, global health is at its beginning stage and recently identified as a drive area. Regarding undergraduate nursing education in India, there is a lack of awareness of the essential global health concept [14]. India currently has 398 medical colleges with a nursing course; among those, some of the universities and schools have just begun to realize the need for introducing Global Health Nursing into the curriculum [15]. Since Global Health Nursing is not embedded Indian nursing council (INC) curriculum for an undergraduate nursing course, this study aims to develop an internal course on Global Health Nursing for nursing students to implement at nursing colleges in India. 

This study adopted the Analysis, Design, Development, Implementation, and Evaluation (ADDIE) model [19] and Gagné’s [20] nine instructional events for the design of each class of Global Health Nursing for Indian nursing students. The ADDIE instructional design model, similar to the nursing process, allows for an interactive systematic continuous evaluation process for course design. This model and its systematic approach allowed instructors to identify and correct the content gaps by feedback easily.

The purpose of this study was (a) to develop an internal elective course on Global Health Nursing for Indian nursing students and (b) to implement and evaluate the course on Global Health Nursing for Indian nursing students.

## 2. Methods and Materials

### 2.1. Methodological Framework

**Figure 1:**
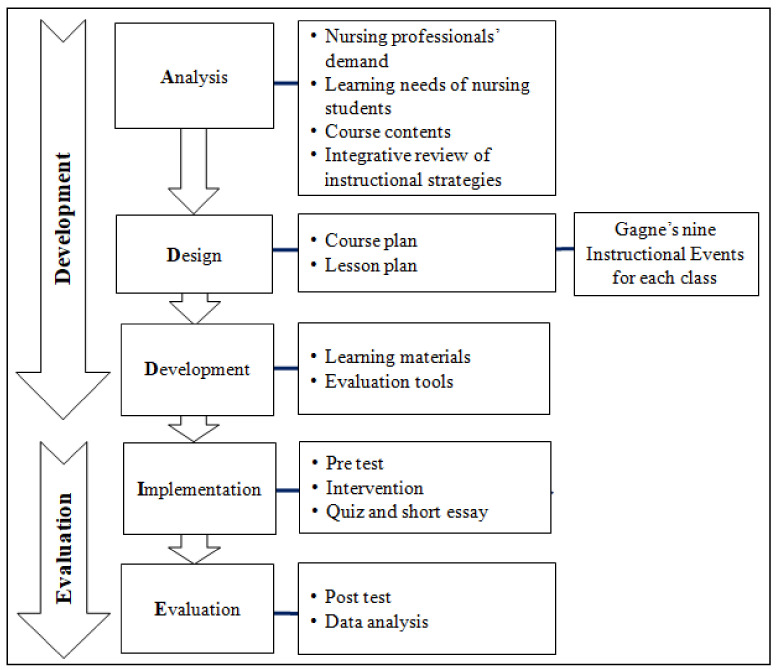
Methodological framework based on ADDIE model.

This study focused ADDIE model with five stages [19] and followed two methods, as shown in Figure 1. In the development of the course on Global Health Nursing, the process was conducted by analysis, design, and development (Table 1). During the evaluation of the course, the process was conducted by implementation and evaluation. Gage’s nine events of instruction [20] were used to design the course (Figure 1).

### 2.2. Development of the Course on Global Health Nursing

#### 2.2.1. Analysis Stage

A four-phase analysis was conducted to develop a Global Health Nursing course for Indian nursing students. The steps were (1) Indian nursing professionals’ demand about the Global Health Nursing course, (2) assessment of learning needs of nursing students (3) course contents of Global Health Nursing, and (4) Instructional strategies for the Global Health Nursing course (Table 1).

##### Indian Nursing Professionals’ Demand

The first phase was conducted through a group interview and discussion with five nursing professionals via online video conferencing (Skype). They were asked to meet for two meetings on a scheduled basis and 2 h/week. The key issues were: (1) What do you think about Global Health Nursing? (2) Why is it necessary to introduce global health into the nursing curriculum? (3) What are the contents to be added, and which grade? (4) Express your personal opinion about the Global Health Nursing course. 

All the professional’s common opinions and comments were coded as five categories: (1) Following WHO standards; (2) Professional nursing issues; (3) Existing common diseases worldwide and prevention methods; (4) International health care system of multidisciplinary teamwork, such as leadership skills, health policies and administration system, health insurance, medical tourism, and traditional methods; (5) Social and environmental aspects of health belief. 

##### Assessment of Learning Needs

Nursing students’ learning needs were analyzed by using the learning needs of the global health survey tool developed by Veras [21]. The survey consists of fourteen items on a 5-point Likert scale (1 = Not at all important to 5 = Extremely important). Nursing students were randomly selected from one of the nursing colleges at the convenience of this study.

The average grade of 3.7 or higher was considered appropriate to meet the learning needs of nursing students. Selected items related to Global Health Nursing domains were part of the global burden of disease, globalization of health and health care, and social and environmental determinants of health.

##### Content of the Course

This study reviewed the well-constructed curriculum of other universities and organizations; the literature was related to Global Health Nursing. The content of the educational course from CUGH, WHO and related literature [21,22,23], were analyzed. Nursing students’ learning needs were analyzed by using the learning needs of the global health survey tool developed by Veras [21]. The survey consists of fourteen items on a 5-point Likert scale (1 = Not at all important to 5 = Extremely important). Nursing students were randomly selected from one of the nursing colleges at the convenience of this study. 

The average grade of 3.7 or higher was considered appropriate to meet the learning needs of nursing students. The final selection of Global Health Nursing domains included the global burden of disease, globalization of health and health care, and social and environmental determinants of health.

##### Instructional Strategies

An integrative review was conducted based on Whittemore and Knalf method [24] to develop the Global Health Nursing course for nursing students. Twenty-four articles were organized to drive and synthesize analytical factors found in these empirical studies. The result of the integrative review showed that the core attributes of teaching–learning strategies include three E-activities: (1) evidence-based learning, (2) experiential learning, and (3) evaluation with reflective learning. 

#### 2.2.2. Design Stage 

The course design was constructed in INC format (Table 2). Domains of Global Health Nursing were designed based on Gagné’s nine instructional events for each class (Table 2).

#### 2.2.3. Development Stage

In the third phase, the course was developed according to the INC protocol, comprising an introduction, placement, hours, course description, course outcomes, instructional strategies, and methods of evaluation. The lesson plan was carefully drawn based on the selected GHC domains by using Gagné’s nine events of instructions. The lesson outcomes were developed for each domain of the class. The lesson outcomes included time, teaching–learning activities, and evaluation methods. Learning materials were reference books, research papers, web articles, PowerPoint slides (maximum 50 slides for each domain), video clips downloaded from YouTube and the global health website that was appropriate for the content, and handouts (relevant lesson contents). Evaluation tools were obtained from existing global health tools and developed open-ended questionnaires to obtain the students’ comments.

### 2.3. Implement and Evaluation of the Course on Global Health Nursing

In this study, the evaluation of the course on Global Health Nursing was followed by two phases of the ADDIE model, such as implementation and evaluation (Table 3). 

#### 2.3.1. Implementation Stage

The course on Global Health Nursing was implemented in July 2017 among an experimental group based on Gagné’s nine Instructional events. The research methods and materials were carefully constructed prior to the intervention as follows.

The study was designed with a quasi-experimental pre-test and post-test with a control group. The study population was fourth-grade nursing students at St. X College of nursing in the academic year 2016–2017 and selected by purposive sampling technique in July 2017. The sample size of fifty participants for 25 in each group needed to meet the requirement for a two-group comparison of intervention study as per Cohen’s guidelines. This study included final grade nursing students in the academic year of 2017. (According to INC regulations, the maximum number of student admission allowed is only 50 students per year. Therefore, according to academic regulation, all 50 final grade-nursing students were selected.) The study excluded first-grade to third-grade nursing students and those who were not willing to participate in this study.

The participants were already divided into two groups according to the regular academic plan, where random selection and assignment were not possible. In this study, twenty-five were assigned to the experimental group (completed clinical practice from February to April 2017), and twenty-five were in the control group (on clinical practice from May to July 2017) at another location. There was no contact between the groups.

#### 2.3.2. Evaluation Stage

This study used the existing survey of Global Health Nursing tool, developed by Veras [21], and the Global Health Nursing for nursing students’ survey [18] to evaluate the effectiveness of the course. Permission was obtained from the original author. At the end of the course, the students’ comments about the course were collected by open-ended questionnaire.

### 2.4. Measurement Scales

#### 2.4.1. General Characteristics 

Age, sex, year of study, place of birth, religion, perception of learning achievement, and prior learning experience of Global Health Nursing education.

The selected variables were ages from 20 to 22 years and grade by year of study. The place of birth was categorized into two, rural and urban, and the religions included Hindu, Muslim, Christian, and Buddhist. The self-perceived academic achievement is categorized into three, high, average, and low, and prior learning experience of Global Health Nursing was considered as “Yes” and “No”.

#### 2.4.2. Knowledge of Global Health 

The survey tool was the self-perceived knowledge of global health developed by Veras and consisted of 16 items on a three-point Likert scale (1 = Not at all, 2 = somewhat and 3 = Good) [21]. Internal consistency of the survey was based on Cronbach’s alpha; the knowledge on Cronbach’s alpha was 0.80. The highest score is considered as having more knowledge about Global Health Nursing.

#### 2.4.3. Skills of Global Health 

The survey tool was the self-perceived skills of global health developed by Veras and consists of 14 items on a 5-point Likert scale (1 = strongly disagree 2 = disagree 3 = neutral 4 = agree and 5 = strongly agree) [21]. While Cronbach’s alpha of the original scale was 0.80, Cronbach’s alpha of this study was 0.81. The highest score is considered as having gained more skills in Global Health Nursing.

#### 2.4.4. Confidence in Performance of Global Health Nursing

The survey tool developed by Wilson et al. [22] was used. The scores of each item were calculated using four points Likert scale (1 = strongly disagree 2 = disagree 3 = agree and 4 = strongly agree). The original scale’s Cronbach 's alpha value was 0.95. In this study, Cronbach’s alpha was 0.90. The highest score is considered as having gained more confidence in the performance of Global Health Nursing.

#### 2.4.5. Students’ Comments

An open-ended questionnaire was used to collect the student’s comments about the course as a qualitative approach according to Bardin’s perspective [25].

### 2.5. Data Collection Methods

In this study, the development and evaluation of the course on Global Health Nursing was conducted at St. X College of nursing in India. The study participants completed a pre-test questionnaire prior to starting the course. The developed course was implemented for 3 weeks (5 h/week) and a 1-h field visit. The field visit was arranged for the students of the experimental group, the students visited Public Health Centre, and a brief lecture was held by global health volunteers and Public Relation Officers of the PHC. After completing the lecture, the experimental group and control group received a post-test survey with the same questionnaire to compare the effectiveness of the developed course on Global Health Nursing. At the end of the course, the open-ended questionnaire was filled out by the intervention group to obtain their opinion and verify the effectiveness of the developed course. 

### 2.6. Data Analysis Methods

The study development and evaluation of the Global Health Nursing course for Indian nursing students was used with SPSS 23.0-win for data analysis. The general characteristics of the participants were analyzed in numbers and percentages. The homogeneity of the control and experimental groups was analyzed with the chi-square test. The differences in the Global Health Nursing survey were analyzed by paired t-tests, t-test, and ANCOVA. An open-ended questionnaire was used to collect the students’ comments about the developed course. The collected qualitative data were analyzed using the content analysis method. According to Patton [21], “content analysis is a process of identifying, coding, and categorizing the primary patterns in the data”. Transcripts were read two or three times thoroughly to prove a sense of the discussion, important keywords and sentences were highlighted. These identified words or sentences were coded by using the terms that emerged directly from the sentence.

### 2.7. Ethical Considerations

The study was conducted according to the guidelines of the Declaration of Helsinki and approved by the Institutional Review Board of I University (2017-02-015-003). In addition, host institution permission was obtained from the chairperson to conduct the Global Health Nursing course (St.Xav. CON/491/16-17). The participants were instructed about the purpose, procedure and methods, their choice to withdraw from the research, and confidentiality.

On requisition of the institution and the intention of this study, the developed course was repeated for the control group as a special lecture and fieldwork at the end of the post-test. This was accomplished by introducing the GHN course to all the nursing students and avoiding internal conflict between the two groups.

## 3. Results

### 3.1. Homogeneity Test for General Characteristics and Outcome Variables

There were no differences in general characteristics between the two groups (*p* > 0.050) (Table 4). The homogeneity test of outcome variables in the pre-test values showed in Table 5. There was no significant difference (t = −1.937, *p* = 0.065) in the pre-test value in knowledge of Global Health Nursing of both groups. According to the results, the skills in the Global Health Nursing pre-test score showed there was a significant difference (t = 3.017, *p* = 0.016) in the experimental group and the control group. The confidence on the performance of the Global Health Nursing pre-test score showed there was no significant difference (t = 0.282, *p* = 0.781) in the experimental group and the control group.

### 3.2. Hypothetical Testing

#### 3.2.1. Hypothesis 1

Knowledge of Global Health Nursing results showed (Table 5) that the post-test mean score of the experimental group was higher than (10.96 ± 5.77) the pre-test score, and there was a statistically significant difference in the experimental group (t = −9.50, *p* < 0.001). The post-test mean score of the control group was lower than (−2.96 ± 3.94) the pre-test score and showed that there was a statistically significant difference in the control group (t = 1.94, *p* < 0.001). The pre- and post-test results of both groups revealed there was a significant difference (t = 8.48, *p* < 0.001). The pre- and post-test mean scores of the experimental groups were higher than the control group. Hence, the hypothesis was accepted.

#### 3.2.2. Hypothesis 2

Skills in Global Health Nursing results showed (Table 5) that the post-test mean score of the experimental group was higher than (14.24 ± 8.78) the pre-test score, and there was a statistically significant difference in the experimental group (t = 8.10, *p* < 0.001). The post-test mean score of the control group was also higher than (1.36 ± 1.72) the pre-test score and showed that there was a statistically significant difference in the control group (t = 3.93, *p* < 0.001). The ANCOVA results showed (F = 96.14, *p* < 0.001) variances of the dependent variable in both groups. The pre- and post-test mean scores of the experimental group were higher than the control group. Hence, the hypothesis was accepted.

#### 3.2.3. Hypothesis 3

Confidence on the performance of Global Health Nursing results showed (Table 5) that the post-test mean score of the experimental group was higher than (7.16 ± 8.78) the pre-test score, and there was a statistically significant difference in the experimental group (t = −4.07, *p* < 0.001). The post-test mean score of the control group was lower than (−2.64 ± 4.13) the pre-test score and showed that there was a statistically significant difference in the control group (t = −3.93, *p* < 0.001). The pre- and post-test results of both the groups revealed there was a significant difference (t = 5.50, *p* < 0.001). The pre- and post-test mean score of the experimental group were higher than the control group. Hence, the hypothesis was accepted.

### 3.3. Students’ Comments

Collected data were carefully categorized as (1) satisfaction of teaching–learning quality and (2) perception of evaluation methods.

#### 3.3.1. Satisfaction of Teaching–Learning Quality

##### Higher Motivation

This category was captured by the following comment on the teaching plan and the quality of teaching: “[it] was actually a good plan to introduce because it also gave us references and supplemental videos to watch, it motivated us to concentrate the class more closey.”

##### Easily Understanding

Students also reported that the Global Health Nursing course improved their understanding (conceptual knowledge) of the complexity and challenges of communication towards collaboration with global health settings. For example, *“[it] really helped me for how I would be ready to talk to a different background client such as language, culture, traditional health practices etc.”*

##### Attractive Teaching–Learning Methods

Students were also in favor of a combination of teaching methods that were used in the Global Health Nursing course that provided an extra supplement to the lectures. In addition, the transformative field experience also facilitated a direct learning experience, which was appreciated by the students: *“The field visit was very interesting because we explored only clinical and community visit, it would be appreciated to add [as an] international course in curriculum”.* However, the international experience and a short-term abroad course are very difficult to add to the curriculum. They were unable to participate on their own because of economic circumstances. All the students mentioned the face-to-face interactions with small group discussions being particularly effective as a method of learning.

#### 3.3.2. Achievement of Learning Outcomes

This section summarizes the achievement of learning outcomes as the perception of the methods of the course evaluation. Evaluation is considered to assess whether learning objectives are met in a developed course. 

##### Completing the Course

In this study, the achievement of lesson outcomes was verified by completing selected evaluation methods such as a weekly report, quiz, group discussion, and presentation. Most of the student comments, the weekly report, and reflective learning and evaluation were interesting to express their opinion about the course: *“The group discussion and presentation were very useful to interact and sharing the ideas within group, but being the end of the course, it was hard to prepare and present report. Felt more difficulty to complete report”.* Students were well aware of their own responsibility for achieving favorable learning outcomes and acknowledged the need to prepare for courses, and their own achievement of the desired outcomes was important.

##### Expression of Own Ability

Students stated that the questionnaire gave an opportunity to show their own learning and the self-development gained while taking the course. Students’ comments revealed that survey tools were too long and would take a long time:*”I think, as a students, if my score a quiz at the end of the course might be low, but after finishing class immediate effect of asking quiz was more effective to express my own capacity.”*

Another student stated that writing a report at the end of each week was appropriate to show their own ability to learn: ” However, if I get ‘good overall but whatever weekly report was ideal’ that would be a particular point I could try to improve on and answer it well in my own”. Another student’s comments stated, “In general, I would give a positive rating for education course in which, I got the feeling to have learned a lot in a pleasant way related to global health nursing”. 

Likert Scale questions received considerably less support, as they were not believed to provide useful information: *“Overall ratings might be easy to analyze statistically but I do not think they really tell you anything”.* Many felt the need to have a clearer outline with progression markers, as one student noted*, “[it] overall used evaluation that show you your progress on education course are really hopeful“.* Students felt that the principal goal of providing individual feedback was helpful to improve teaching skills; therefore, free text comments were preferred. 

At the end of the course, the control group also received a special lecture about Global Health Nursing. Overall, the participants gave valuable insight in terms of potential improvement to the delivery of the Global Health Nursing course content. 

## 4. Discussion

### 4.1. Development of the Course on Global Health Nursing

In this study, the development of the course on Global Health Nursing carefully followed the ADDIE model [19]. In the first phase, nursing professionals’ demand, the learning needs of nursing students, course content, and a modified framework integrative review of instructional strategies were analyzed. The findings of the integrative review and systematic analysis with experts’ opinions were clearly explained that the development of Global Health Nursing is one of the great opportunities for developing countries facing the current diverse international changes [22,26,27]. Therefore, it is necessary to investigate whether the education contents were reflected in the nursing curriculum. 

The second phase was course design; numerous studies noted that the courses of Global Health Nursing were considered as an extracurricular subject rather than the regular course [28,29,30,31,32,33]. Therefore, this study also followed the same and was designed to have a duration of 16 h within 3 weeks among the fourth-grade nursing students as an elective course. Another similar study [28] was conducted for 3 weeks among senior nursing students entitled to an ongoing educational program to enhance the globalization of nursing. A biannual survey developed by the Section of Review Council found that 88% of family nursing courses have some formal global health curriculum, and most schools devote less than 10 h to global health during their training [29,30,31,32]. 

Our findings reported that the field visit was very useful for self-learning by seeing or performing by learning approach to improve the knowledge and skills themself. The importance of self-learning was notified in a survey of the global health curriculum in 17 Canadian health professional schools that found a growing demand for global health training, but the training courses were not responding satisfactorily [30,33]. Therefore, The Canadian Nurses Association recognized the need to develop nursing leadership in global health courses [30]. It is more necessary to plan for the achievement of the course objectives within a limited time duration. In order to overcome time limitations, Global Health Nursing-based education was found to be equally effective in both didactic and self-learning approaches [31]. 

The third-phase course was developed by selected domains of Global Health Nursing with learning materials, selection of appropriate instructional strategies, and evaluation methods. Allocated creative projects and skilled teaching–learning activities are essential for developing course-related global health [22,26,27]. Some studies noted effective teaching skills to be developed for broadening global health perspectives [31,32,33,34,35]. Other studies also insisted that students could gain awareness of Global Health Nursing issues and contribution by continuing education based on their experiences [32,33,34,35,36]. Therefore, it is good news that the global and public health program at Manipal University in India covered the international aspects of environmental health, including weekly field-based practicum and implementation of various WHO programs at respective levels [13,14,15]. 

### 4.2. Implementation and Evaluation of the Course on Global Health Nursing

In this study, quantitative and qualitative questions were surveyed for evaluating the course after implementation. At the end of the course on Global Health Nursing, feedback was received through questionnaires including knowledge of global health, skills of global health developed by Veras [21], and confidence on performance of Global Health Nursing developed by Wilson et al. [22]. Moreover, an open-ended questionnaire about the developed course was distributed to obtain the students’ comments for the intervention group. From the results, the knowledge, skills, and performance of the experimental group were revealed to be significantly higher than the control group. 

The study results for knowledge in global health showed that more than 70% of the study participants reported language barriers and adverse impacts on health and health care. According to the literature, one of the barriers to effective health care services is language; it reported that multi-linguistic knowledge and skills help nursing students to provide effective care for their clients [26,27,28]. Nursing students gained average skills in the global health activity of working with different backgrounds, understanding perspectives, and accessing resources to keep up to date with global health issues; this was consistent with other studies that revealed study participants had gained average skills in awareness of the health services available in different backgrounds and effectiveness in completing clinical responsibilities [26,27,28,29,30,31,32,33,34,35]. Another study reported team disagreements related to care for patients with different backgrounds; difficulties identifying the needs of patients with different backgrounds showed lack of understanding among nursing students [36]. The same results were explained by WHO, which also recommends admission policies to enroll students with a rural background in order to increase the probability of these students developing their practice in rural areas and different backgrounds [3,6,11]. 

The students’ confidence in the performance of Global Health Nursing was higher in politics, economics, and nursing professional issues in this study. The various studies stated that Global Health Nursing training is important because it improves the participant’s communication, social, and economic skills, and it enables them to practice in diverse environments [36,37,38]. The findings supported that nursing students showed good communication skills score even though language barrier issues [39,40]. A study noted one of the barriers to effective health care service was language. In addition, multi-linguistic knowledge and skills help health professionals to provide effective performance in Global Health Nursing [31,36,38]. Therefore, communication skills and language problems should be trained simultaneously in the Global Health Nursing course.

From the qualitative results of the present study, participants of experimental students described that they were satisfied with the teaching–learning quality and achievement of their learning goals. A study of the Global Health Nursing course should gauge teaching quality by addressing various areas such as the content taught, teacher characteristics, and, most importantly, learning outcome. Students were encouraged to pursue their careers and profession with the vision of being a leader, and this result was found in many previous studies [35,36,37,39,40]. Several studies have clarified Global Health Nursing, implemented by quantitative and qualitative data analysis methods [27,28,29,32,33,34]. Bozorgmehr et al. emphasized that the Global Health Nursing education framework should also consider building on existing strengths in the country in terms of public health delivery, leadership, systems, and training [38]. Overall, students gave valuable insights in terms of potential improvements to the delivery of the content of this Global Health Nursing course and highly recommended to plan a similar course to attain the objective of Global Health Nursing in four years of nursing course.

This study has some limitations; it was focused on the development and evaluation of a course on Global Health Nursing for Indian nursing students. The findings have an inherent limitation in generalization because all respondents were only female, and the majority of them were Hindu and Christian. Further research should focus on both the gender and Muslim students. In addition, the course should be implemented in various settings, such as rural and urban colleges of nursing in each state of India. 

## 5. Conclusions

The purpose of this study was to develop and evaluate the course on Global Health Nursing for Indian nursing students. The course on Global Health Nursing was developed based on the ADDIE model and designed using Robert Gagné’s nine events of instruction. The findings of this study support evidence that the incorporation of Global Health Nursing education-based competency into nursing education curricula enhances the level of knowledge and skills of global health competence among undergraduate nursing students. The suggestions expressed for future study regarding Global Health Nursing courses are the following: various regions with a large sample size of nursing students would be preferred to obtain perfect effectiveness of course; long-term field experience to enhance global student health-oriented cultural competence; development of evaluation tools for checking trans cultural skill; and training related to Global Health Nursing competency for nursing educators, and nurses should be considered.

## Figures and Tables

**Table 1:** Development of the Course on Global Health Nursing.

Phase of Course Development		Research Process	
Analysis	▪ Nursing professionals demand ▪ Learning needs of students ▪ Course contents ▪ Integrative review of instructional strategies	▪ Interviewed five nursing professionals ▪ Analyzed learning needs for nursing students ▪ Selected contents of global health domains derived from existing CUGH and WHO ▪ Reviewed existing articles and analyzed the teaching–learning strategies	
Design	▪ Course plan ▪ Lesson plan	▪ Prepared course plan based on INC format ▪ Designed weekly lesson plan based on Gagné’s nine events of instruction in each class.	
Development	▪ Learning materials ▪ Evaluation tools	▪ Prepared PPT slides, handouts, and video clips. ▪ Selected existing tools of Global Health Nursing and obtained permission from the original author.	

CUGH—Consortium of Universities for Global Health, WHO—World Health Organization, INC—International Nursing Council.

**Table 2:** Comparison of Global Health Nursing Domains.

CUGH and WHO	Wilson and Ventura	INC	Selected
Global burden of disease	Global burden of disease	--	Global burden of disease.
Globalization of health and health care.	Globalization of health and health care	--	Globalization of health and health care.
Social and environmental determinants of health	Social and environmental determinants of health	--	Social and environmental determinants of health
Capacity strengthening	Health implications of migration, travel, and displacement	Capacity strengthening	--
Collaboration, partnering, and communication	--	Community health nursing	--
Ethics	Healthcare in low-resource settings	Fundamentals of nursing	--
Health equity and social justice	--	Health equity and social justice	--
Course management	--	Nursing management	--
Socio cultural and Political awareness	--	Sociology	--
Strategic analysis.	--	Strategic analysis	--

CUGH—Consortium of Universities for Global Health, WHO—World Health Organization, Wilson et al. [22] and Ventura et al. [23], INC—Indian Nursing Council.

**Table 3:** Evaluation of the Course on Global Health Nursing.

Stage	Research Process	Research Process
Implementation	▪ Pre-test ▪ Intervention ▪ Quiz and short essay	▪ Conducted pre-test with selected survey tool ▪ Implemented an internal course to experimental group ▪ Conducted weekly after completion of each domain
Evaluation	▪ Posttest ▪ Data analysis	▪ Survey tool and students’ comments. ▪ Chi-square test, paired *t*-test, *t*-test, and ANCOVA with SPSS

**Table 4:** The Homogeneity Test of General Characteristics of Nursing Students (N = 50).

Characteristics	Categories	Exp. (n = 25)	Con. (n = 25)	F/χ ^2 *^	*p*
		n (%)	n (%)		
Age (Years)	20	3(12)	5(20)	2.10	0.350
	21	21(84)	19(76)		
	22	01(4)	1(4)		
Religion	Hindu	24(96)	22(88)	1.08	0.297
	Christian	01(4)	3(12)		
Perception of learning achievement level	High	4(16)	2(8)	7.09	0.281
	Average	18(72)	22(88)		
	Low	3(12)	1(4)		
Prior experience of Global Health Nursing	Yes	10(40)	11(44)	0.99	0.434
	No	15(60)	14(56)		

* Fisher’s exact test or Chi-square test; Exp—Experimental group; Con—Control group.

**Table 5:** Hypothetical Testing (N = 50).

Variables	Group	Pre Test	Post Test	Difference	t/F (*p*)
		Mean ± SD		(Post–Pre)	
Knowledge	Experiment	29.24 ± 4.37	40.20±3.45	10.96 ± 5.7	8.48 < 0.001
		t = −9.50 (*p* < 0.001)			
	Control	31.48±4.58	28.52±3.95	−2.96 ± 3.94	
		t = −1.94 (*p* < 0.001)			
Skills	Experiment	40.20±3.45	54.44±8.45	14.24 ± 8.78	96.14 < 0.001
		t = 8.10 (*p* < 0.001)			
	Control	47.16±8.07	48.80±8.21	1.36 ± 1.72	
		t = 3.93 (*p* < 0.001)			
Performance on Global Health Nursing	Experiment	92.88±8.58	100.04±5.91	7.16 ± 8.78	5.50 < 0.001
		t = −4.07 (*p* < 0.001)			
	Control	92.12±12.34	89.48±11.60	−2.64 ± 4.13	
		t = −3.93 (*p* < 0.001)			

## Data Availability

The raw data supporting the conclusions of this article will be made available by the authors without undue reservation.
